# Day/night temperature fluctuations delay the high-temperature-induced decline in Cry1Ac endotoxin concentration in cotton squares

**DOI:** 10.3389/fpls.2026.1913082

**Published:** 2026-08-27

**Authors:** Yuting Liu, Run Ou, Ziyi Gu, Wenkai Gao, Yuan Chen, Xiang Zhang, Dehua Chen

**Affiliations:** Jiangsu Key Laboratory of Crop Genetics and Physiology/Co-Innovation Center for Modern Production Technology of Grain Crops, Yangzhou University, Yangzhou, China

**Keywords:** Bt cotton, cotton square, day/night temperature fluctuation, high temperature stress, nitrogen metabolism

## Abstract

**Introduction:**

High-temperature stress significantly impacts Cry1Ac endotoxin efficacy in *Bacillus thuringiensis* (Bt) cotton, yet the effects of realistic day/night temperature fluctuation remain unclear.

**Methods:**

Hence, a 2-year (2021-2022) plot experiment was carried out to investigate the response of square Cry1Ac endotoxin concentration to fluctuating high temperature (HDT/NNT, 38/27 °C) compared with a control treatment (CK, 32/25 °C) in cultivars ‘Sikang 1’ and ‘Sikang 3’.

**Results:**

The results indicated that the Cry1Ac endotoxin concentration remained stable under CK but decreased significantly under HDT/NNT only after 7 days of stress, with the decline intensifying over time. In 2021, the reduction was 14.18% and 30.88% on the 7th day and 10th day for S1, respectively, and 11.32% and 25.21% for S3. Physiologically, this reduction coincided with decreased soluble protein content and glutamate-oxaloacetate transaminase and glutamic-pyruvic transaminase activities, alongside increased free amino acid content and protease and peptidase activities.

**Discussion:**

We hypothesize that normal night temperatures provide temporary physiological compensation against daytime high-temperature stress damage, maintaining a dynamic balance between Cry1Ac synthesis and degradation. However, as high-temperature stress persists between 4 and 7 days, the cumulative damage exceeds the nighttime compensatory capacity limit, which quantitatively and qualitatively disrupts this metabolic equilibrium by day 7, accelerating endotoxin loss. In summary, prolonged high-temperature stress (approximately 1 week) at the peak square stage in the field environments requires close monitoring and timely agricultural interventions.

## 1 Introduction

Cotton (*Gossypium hirsutum* L.) is recognized as one of the world’s most dominant industrial crops (Jehangir, 2023). It is known as a warm-season annual fiber crop of tremendous importance to trade and the economy. However, cotton growers suffered from severe losses caused by bollworm (*Helicoverpa armigera* and *Helicoverpa zea*) infestations (Legan et al., 2024; Holman et al., 2025; Yue et al., 2025). Increased populations of bollworms have been reported to significantly reduce cotton yields. Moreover, there was a highly positive correlation between the number of bollworm larvae and the number of infested squares, with one second-generation larva being capable of damaging an average of 7.4 squares (Baishan et al., 2000). Because the Cry1Ac endotoxin expressed by the Bt gene in transgenic *Bacillus thuringiensis* cotton (Bt cotton) has shown an effective lethal effect on bollworms (Liu et al., 2001; Héma et al., 2009), Bt cotton has now been widely adopted by cotton growers worldwide (Rout et al., 2023). The spread of Bt cotton, a major agricultural revolution in biotechnology, has not only increased growers’ incomes but also reduced the use of chemical pesticides (Gould, 1988; Gasser and Fraley, 1989). Most importantly, the fiber quality of Bt cotton does not differ from the non-transgenic varieties (Gasser and Fraley, 1989; Wu, 2007).

However, due to the fluctuation of Cry1Ac endotoxin content in Bt cotton, the insecticidal effect it presents is unstable (Zhao et al., 2000; Liu et al., 2022). Therefore, research on how to solve this problem has become a topic of great importance. Temporally, the maximum value of Cry1Ac endotoxin concentration tends to appear in the early growth stage; however, as the plant grows, this concentration shows a decreasing trend, reaching its minimum during the yield formation period which consequently decreases insect resistance (Adamczyk and Meredith, 2004; Chen et al., 2018). Additionally, there are significant differences in Cry1Ac endotoxin concentrations among different organs: The concentration in vegetative organs is higher than that in reproductive organs, and the concentration in younger organs is greater than that in mature organs (Liu et al., 2019, Liu et al., 2024). It has also been revealed that the Cry1Ac endotoxin concentration in Bt cotton is related to nitrogen content and that the higher the nitrogen content of organs in Bt cotton, the higher the bollworm mortality rate is (Saini and Dhawan, 2014).

Apart from the plant itself, the Cry1Ac endotoxin concentration is also susceptible to numerous extreme environmental factors (Wang et al., 2009; Zhou et al., 2019). These stressors include extreme high and low temperatures, drought, waterlogging, air humidity, and soil salt stress (Luo et al., 2008; Chen et al., 2012a, Chen et al., 2014; Wang et al., 2018; Chen et al., 2023). Among the multiple factors, high temperature is one of the most influential factors resulting to the instability of Cry1Ac endotoxin content (Zhang et al., 2018). The damage of high temperatures extends beyond this; most studies have shown that high temperatures lead to a reduction in boll number and boll weight, which is highly correlated with decline in cotton yield (Wang et al., 2017; Gao et al., 2021). Furthermore, the current trend of global warming is going to persist. According to the IPCC (Calvin et al., 2023), the global surface temperature from 2011 to 2020 is approximately 1.1 °C higher than that during the period 1850–1900, which has led to frequent extreme weather events in recent years. Worse still, the current trend of global warming is going to persist, with global warming likely to reach 2.2 °C-3.5 °C by 2100. Moreover, the frequency and intensity of extreme heat events will further increase with every 1 °C increase in temperature. Therefore, investigating the response of the Cry1Ac endotoxin concentration in Bt cotton under high-temperature conditions is crucial for the sustainable development of cotton production.

Previous studies demonstrate that high-temperature stress generally reduces Cry1Ac endotoxin levels in Bt cotton, although the severity heavily depends on the specific organ and growth stage. For instance, continuous exposure to 39 °C significantly lowered toxin concentrations in boll shells (Heng et al., 2016), and temperatures exceeding 38 °C caused squares to lose their insect resistance (Chen et al., 2014). Interestingly, main stem leaves appear more vulnerable to heat during the boll-setting stage than during earlier reproductive phases (Chen et al., 2005). While heat-induced toxin reduction is reversible once plants return to cooler conditions—with the required recovery time directly proportional to the stress duration (Chen et al., 2013)—these foundational findings predominantly rely on constant-temperature models that fail to capture natural diurnal fluctuations. To better reflect field environments, more recent work has utilized alternating day/night temperature cycles, such as evaluating peak-stage boll shells under a 38/27 °C regime (Zhang et al., 2018). Yet, this methodological shift has largely overlooked early reproductive organs. Squares at the peak squaring stage are physiologically much more sensitive to environmental stress and prone to abscission compared with mature boll shells. Although we know that a drop in temperature can trigger metabolic recovery (Chen et al., 2013), it remains entirely unclear whether natural nighttime relief is sufficient to maintain Cry1Ac equilibrium in squares during prolonged, multiday heatwaves. This critical knowledge gap ultimately weakens our ability to accurately predict early-season insect resistance in the field.

To address this limitation, this study was carried out to investigate the response of square Cry1Ac endotoxin concentrations and corresponding nitrogen metabolic profiles to a realistic 38/27 °C day/night fluctuation during the peak square stage. The primary innovation of this work lies in decoding the dynamic balance between daytime protein degradation and nighttime metabolic recovery specifically within square (the reproductive organ) over an extended stress duration, distinguishing it from prior studies on constant-temperature or late-stage boll shell. We hypothesized that normal nighttime temperatures offer physiological compensation that counteracts daytime heat stress damage and influences the Cry1Ac endotoxin concentration. Accordingly, the main objective of this experiment was to investigate the response of squares to high-temperature stress under the day/night temperature fluctuation and to investigate the physiological mechanism behind it. These findings provide a more accurate, square-stage-specific framework for optimizing pest management in Bt cotton under changing climatic conditions.

## 2 Materials and methods

### 2.1 Experimental design

The experiment was carried out in 2021–2022 at Yangzhou University, Yangzhou, China (32°30’N, 119°25’E), using pot planting. Two medium maturity Bt cotton cultivars, ‘Sikang 1’ (conventional cultivar) and ‘Sikang 3’ (hybrid cultivar), were sown into the greenhouse on April 16 in both years. Seedlings were transplanted into pots on May 22 at 35 days after sowing (DAS). Each porcelain pot measured 50 cm in height and 40 cm in diameter, with a volume of 62.8 L. Soil for potting is sandy loam soil (containing 1.96 g kg^–1^ organic matter, and available N, P, and K at 113, 36.7, and 80.4 mg kg^–1^, respectively) that has been naturally dried and sifted to remove impurities, and then compacted with water. Each cultivar was transplanted in 48 pots, and one seedling was transplanted per pot. A dynamic irrigation strategy was implemented throughout the growing season. Soil moisture of each pot was rigorously monitored three times every day (morning, noon, and evening) by using a soil moisture/salinity/temperature tri-parameter (WET-2 Soil Sensor, Delta-T Devices, UK). Based on these real-time measurements, irrigation was applied as needed to strictly maintain the soil water content at 75% of the maximum field capacity in the pots throughout the experimental period. All plants were fertilized according to local management. ‘Sikang 1’ and ‘Sikang 3’ flowered at 89 and 91 DAS, first boll opened on 135 and 138 DAS, respectively.

The experiment for each cultivar was arranged in a completely randomized design, consisting of two temperature treatments: the control check (CK, 32/25 °C) and the fluctuating day/night temperature treatment with high daytime temperature/normal night temperature (HDT/NNT, 38/27 °C). In the HDT/NNT treatment, plants were exposed to a threshold temperature of 38 °C from 6:00 a.m. to 6:00 p.m., followed by 27 °C for the remaining night hours. Similarly, in the control treatment plants were exposed to a threshold temperature of 32 °C from 6:00 a.m. to 6:00 p.m., followed by 25 °C for the remaining night hours. A total of 24 pots of each treatment of each cultivar separately were moved to the climate chambers to start the treatments at the peak square stage and lasted for 10 days. In all climate chambers, the daytime (6:00 a.m. to 6:00 p.m.) conditions were regulated at a photon flux density of 200 μmol m^-^² s^-^¹ and 70% relative humidity. Before the plants were moved to the climate chambers, the squares of the first fruiting node of the fifth to eighth fruiting branches that appeared for 15, 11, 8, and 5 days were individually labeled to ensure that all squares picked across different sampling dates were at an identical physiological developmental stage (15-day-old squares).

### 2.2 Preparation of plant material

In 2021 and 2022, 16 labeled 15-day-old squares from different plants of each treatment were collected at 11:00 a.m. on 0, 4, 7, and 10 days after the start of treatments. Each cotton square was collected from different plants. The collected samples were immediately frozen by liquid nitrogen and then stored in an ultra-low-temperature refrigerator and used for subsequent indicator measurements. Before testing, the 16 samples were mixed together and then divided into three biological replicates for measurement.

### 2.3 Measurements

#### 2.3.1 Cry1Ac endotoxin concentration

The square Cry1Ac endotoxin concentration was assayed by immunological analysis ELISA (Chen et al., 1997). Three subsamples were excised from each square using a standard 2-cm-diameter paper ticket punch. Square tissue extracts (ca. 0.5 g) were prepared by homogenizing the frozen tissue in 2 mL extraction buffer (Na_2_CO_3_ 1.33 g, DTT 0.192 g, NaCl 1.461 g, and Vitamin C 0.5 g dissolved in 250 mL distilled water) then transferring to a 10-mL centrifuged tube; the residue remaining on the wall of the mortar was washed again with 3 mL of the buffer, and this was also added to the centrifuged tube. The contents of this tube were shaken with hand and stored at 4 °C for 4 h. The supernatants were collected after centrifugation at 10,000 × g at 4 °C for 20 min, passed through a C18 Sep-Pak cartridge (Waters, Milford, MA), and the three subsamples were pooled for determination. Quantification of the Cry1Ac in the combined samples was conducted using a commercially available kit (Scientific Service, Inc., China Agriculture University, Beijing). Microtitration plates were coated with the standard Cry1Ac insecticidal proteins and samples, incubated at 37 °C for 4 h. Antibodies were added to each well and incubated for further 30 min at 37 °C. The antibodies against the Cry1Ac insecticidal protein were obtained as described by Weiler et al. (1981). Then, horseradish peroxidase-labeled goat anti-rabbit immunoglobulin was added to each well and incubated for 30 min at 37 °C. Finally, the buffered enzyme substrate (orthophenylenediamine, at a concentration of 2mg mL^–1^, 100 μL per well) was added, and the enzyme reaction was carried out in the dark at 37 °C for 15 min and then terminated using 3 mol L^–1^ H_2_SO_4_. The absorbance was recorded at 490 nm. The ELISA standard curve was established as y = 0.8467x–1.5251 (R² = 0.9956), where y is the logit value of the absorbance, and x is the logarithm of the Cry1Ac insecticidal protein concentration. The linear range of the standard curve was 10–200 ng mL^–1^. The limit of detection was determined to be 9.14 ng mL^–1^. The recovery rate range was 89.42%–97.95%.

#### 2.3.2 Soluble protein and free amino acid content

The square samples (0.5 g) were used for the extraction and analysis of soluble protein and free amino acid content. The samples were homogenized at 4 °C in 3 mL cold water (Milli-Q reagent grade) and centrifuged at 800 × g for 5 min. The supernatant was stored on ice. The total free amino acid content was determined by the ninhydrin assay according to Yemm and Cocking (1955). The total soluble protein content was determined by the Coomassie Blue dye-binding assay of Bradford (1976). The absorbance readings were converted into protein concentrations using bovine serum albumin in the standard curve.

#### 2.3.3 Glutamate-oxaloacetate transaminase and glutamic-pyruvic transaminase activities

The square samples (0.5 g) were homogenized in buffered medium (0.05 mmol L^–1^ Tris–HCl, pH 7.2), and the homogenate was centrifuged at 26 100 × g for 10 min at 0 °C. The supernatant was analyzed for glutamate-oxaloacetate transaminase (GOT) activity. A mixture of 0.5 mL of a 0.8-mol L^–1^ aspartate in 0.1 mol L^–1^ Tris–HCl (pH 7.5) + 0.1 mL of a 2-mmol L^–1^ pyridoxal phosphate solution was used, and to this 0.2 mL of a 0.1-mol L^–1^ 2-oxoglutarate solution and 0.2 mL the enzyme preparation were added, the reaction mixture was incubated at 37 °C for 10 min followed by termination of reaction with 0.1 mL of a 0.2-mol L^–1^ trichloroacetic acid solution, and then the pyruvate with chromogen was converted to pyruvate hydrazone. The color intensity of the hydrazone in saturated water toluene was measured at 520 nm. The GOT activity, in terms of pyruvate production, was calculated from authentic pyruvate standards run simultaneously. The procedure used for assaying the activity of glutamic-pyruvic transaminase (GPT) was identical to that described for the GOT assay except that in the GPT assay, 0.5 mL of a 0.8-mol L^–1^ alanine in 0.1 mol L^–1^ Tris–HCl (pH = 7.5) were substituted for a 0.5-mL of a 0.1-mol L^–1^ buffered aspartate solution in the reaction mixture and aniline citrate addition was omitted (Wu et al., 1998).

#### 2.3.4 Protease and peptidase activities

The samples (0.8 g) were homogenized at 4 °C in 1 mL of β-mercaptoethanol extraction buffer (a mixture of ethylene glycol, sucrose, and phenylmethylsulfonyl fluoride pH = 6.8). Cell debris were removed by centrifugation, and the supernatant was placed on ice and immediately used to estimate the square protease. Protease activity was determined using azocasein as substrate (Vance et al., 1979). The reaction mixture contained 0.4 mL of 10 mg mL^–1^ azocasein, 0.4 mL of 0.05 M succinate buffer (pH = 5.5) containing 10 mM mercaptoethanol, and 0.2 mL of nodule cell-free extract. After 1 h at 35°C, the reaction was stopped by adding 1 mL of 1 N perchloric acid. The precipitated protein was removed by centrifugation at 8,000 × g for 15 min, and the absorbance of the supernatant was read at 400 nm expressing as change in absorbance.

For peptidase activity determination, the square samples (0.5 g) were homogenized at 4 °C in 8 mL of Tris–HCl extraction buffer (a mixture of 4 mmol L^–1^ DTT, 4 mmol L^–1^ EDTA, 1% PVP, pH = 7.5) and then centrifuged at 15,000 × g for 30 min at 0 °C. The supernatant was used to estimate the peptidase activity (Wittenbach, 1979): A mixture of 0.4 mL acetate buffer (pH = 4.8), 1% bovine hemoglobin compounded with 0.2 mL acetate buffer (pH = 4.8) (final concentration 0.2%), was incubated at 37 °C for 10 min, and then 0.4 mL the enzyme sample was added and was incubated at 38 °C for 60 min followed by termination of reaction with 1 mL of a 10% trichloroacetic acid solution (the control was added to the termination solution before the reaction), centrifuged at 4,000 × g for 5 min again after being motionless at 4 °C for 30 min. The supernatant was pooled for analysis of the amino acid content, which was determined by ninhydrin assay (Yemm et al., 1955), and expressed as change in absorbance.

### 2.4 Data processing and statistics

Experimental data were analyzed using SPSS 26 (IBM Corp., Armonk, NY, USA). A one-way ANOVA was performed. Before the ANOVA was conducted, all data were tested for normality using the Shapiro–Wilk test and for homogeneity of variances using Levene test. Pairwise comparisons between different durations were performed using least significant difference (LSD) test at *P* ≤ 0.05 and *P* ≤ 0.01. Correlation coefficients among the physiological indicators were calculated using simple Pearson linear correlation analysis without multiple comparison correction. The figures were plotted with Origin 2021 (OriginLab, Northampton, Massachusetts, USA). Furthermore, three-factor ANOVA was performed to evaluate all main effects and interaction terms (Cultivar × Treatment, Cultivar × Time, Treatment × Time, and Cultivar × Treatment × Time).

## 3 Results

### 3.1 Response of Cry1Ac endotoxin concentrations to high temperature

Three-way ANOVA showed that the Cry1Ac endotoxin concentration was significantly affected by cultivar, temperature treatment, and sampling time in both 2021 and 2022 (*P ≤* 0.01; **Table 1**). Importantly, a strong Treatment × Sampling time interaction was observed in both years (*P ≤* 0.01), showing that the temperature effect was strongly time-dependent. Minor cultivar × Treatment and cultivar × Sampling time interactions were detected in 2021 (*P* = 0.03), but not in 2022 (*P* > 0.05). The three-way interaction was non-significant in both years (*P* > 0.05), indicating that the temporal response of Cry1Ac protein to high-temperature stress was consistent across cultivars.

**Table 1:** Three-way ANOVA of cultivar, treatment, and sampling time on Cry1Ac endotoxin concentration in 2021 and 2022.

Source of variation	2021		2022	
	*F* value	*P* value	*F* value	*P* value
Cultivar (C)	14.10**	≤ 0.01	25.55**	≤ 0.01
Treatment (T)	584.86**	≤ 0.01	654.22**	≤ 0.01
Sampling time (S)	180.74**	≤ 0.01	327.2**	≤ 0.01
Cultivar × treatment	5.12*	0.03	0.66	0.42
C × S	3.52*	0.03	0.73	0.54
T × S	225.84**	≤ 0.01	285.4**	≤ 0.01
C × T × S	2.37	0.09	0.91	0.45

* and ** indicate significant differences at the *P ≤* 0.05 and *P ≤* 0.01 probability levels, respectively.

Square Cry1Ac endotoxin concentrations did not change significantly from the 0th to 10th days under control treatment for both cultivars. In contrast, under HDT/NNT, the Cry1Ac endotoxin concentration remained stable at 0 and 4 days of stress duration, but there was a significant decrease in Cry1Ac endotoxin concentration at 7 days of stress duration, and the decline intensified with prolonged stress duration (**Table 2**). Compared with the control, the Cry1Ac endotoxin concentration reduced by 14.18% and 30.88% on the 7th day and 10th day, respectively, for S1, and those values were 11.32% and 25.21% for S3 in 2021. In 2022, the reduction on the 7th day were 10.68% and 26.94% for S1 and 11.87% and 25.24% for S3, respectively. In addition, the variations in S1 was greater than that in S3.

**Table 2:** Effects of high-temperature stress under day/night temperature fluctuation on the square Cry1Ac endotoxin concentration in 2021 and 2022 (ng·g^-1^ FW)^1)^.

Stress time	S1^2)^				S3^2)^			
	2021		2022		2021		2022	
	CK^3)^	HDT/NNT^3)^	CK	HDT/NNT	CK	HDT/NNT	CK	HDT/NNT
0 days	527.32 ± 4.94aA	525.64 ± 8.24aA	532.03 ± 3.13aA	528.32 ± 5.08aA	548.5 ± 11.49aA	545.74 ± 8.66aA	554.04 ± 7.72aA	556.97 ± 8.69aA
4 days	531.64 ± 3.62aA	527.93 ± 10.25aA	532.04 ± 10.87aA	528.7 ± 7.63aA	543.95 ± 3.32aA	539.25 ± 10.26aA	547.15 ± 4.93aA	551.45 ± 6.13aA
7 days	536.73 ± 4.75aA	460.64 ± 4.56bB	539.66 ± 5.53aA	478.55 ± 7.84bB	539.14 ± 10.43aA	481.55 ± 10.37bB	552.14 ± 4.15aA	486.62 ± 5.85bB
10 days	535.25 ± 7.34aA	369.95 ± 5.32cC	541.15 ± 11.01aA	404.75 ± 8.57cC	537.63 ± 9.06aA	392.77 ± 5.75cC	549.66 ± 3.61aA	410.93 ± 5cC
*F* value	1.47	263.39**	1.78	236.54**	0.76	215.28**	1.19	314.09**
*P* value	0.31	0.01	0.25	≤ 0.001	0.56	≤ 0.01	0.39	≤ 0.01

^1)^ FW means fresh weight.

^2)^ S1 and S3 represent cultivars ‘Sikang 1’ and ‘Sikang 3’, respectively.

^3)^ CK represents the control treatment; HDT/NNT represents the fluctuating day/night temperature treatment with high daytime temperature/normal night temperature (38/27 °C).

Different lowercase and uppercase letters in each column represent significant differences at the P ≤ 0.05 and P ≤ 0.01 probability levels, respectively.

** indicates significant difference at the P ≤ 0.01 probability level.

Pairwise comparisons between different durations were performed using least significant difference (LSD) test at *P* ≤ 0.05 and *P* ≤ 0.01. Different lowercase letters at each column represent significant difference between duration at *P* ≤ 0.05 under the same treatment for the same cultivar. Different uppercase letters at each column represent significant difference between duration at *P* ≤ 0.01 under the same treatment for the same cultivar.

### 3.2 Response of square soluble protein and free amino acid contents to high temperature

Three-way ANOVA showed that soluble protein content was significantly affected by cultivar, temperature treatment, and sampling time in both 2021 and 2022 (*P ≤* 0.01; **Table 3**). Importantly, a strong Treatment × Sampling time interaction was observed in both years (*P ≤* 0.01), showing that the temperature effect was strongly time-dependent. Significant Cultivar × Treatment and Cultivar × Sampling time interactions were detected in 2021 (*P ≤* 0.05), but not in 2022 (*P* > 0.05). The three-way interaction was significant in 2021 (*P ≤* 0.01) but non-significant in 2022 (*P* > 0.05).

**Table 3:** Three-way ANOVA of cultivar, treatment, and sampling time on square soluble protein content in 2021 and 2022.

Source of variation	2021		2022	
	*F* value	*P* value	*F* value	*P* value
Cultivar (C)	198.39**	≤ 0.01	432.15**	≤ 0.01
Treatment (T)	186.21**	≤ 0.01	187.73**	≤ 0.01
Sampling time (S)	51.68**	≤ 0.01	79.58**	≤ 0.01
Cultivar × treatment	5.50*	0.03	3.21	0.08
C × S	6.85**	≤ 0.01	0.40	0.76
T × S	70.54**	≤ 0.01	94.91**	≤ 0.01
C × T × S	6.03**	≤ 0.01	0.05	0.98

* and ** indicate significant differences at the *P ≤* 0.05 and *P ≤* 0.01 probability levels, respectively.

A largely comparable ANOVA profile was also observed for free amino acid accumulation (**Table 4**). Specifically, free amino acid content was significantly affected by cultivar, temperature treatment, and sampling time in both 2021 and 2022 (*P ≤* 0.01; **Table 3**). Importantly, a strong Treatment × Sampling time interaction was observed in both years (*P ≤* 0.01), showing that the temperature effect was strongly time-dependent. A significant Cultivar × Sampling time interaction was detected in both years (*P ≤* 0.01), whereas the Cultivar × Treatment interaction was significant only in 2022 (*P ≤* 0.01). The three-way interaction was significant in both years (*P ≤* 0.05), indicating minor cultivar-dependent variations in the temporal accumulation of free amino acids under high-temperature stress.

**Table 4:** Three-way ANOVA of cultivar, treatment, and sampling time on square free amino acid content in 2021 and 2022.

Source of variation	2021		2022	
	*F* value	*P* value	*F* value	*P* value
Cultivar (C)	207.76**	≤ 0.01	169.64**	≤ 0.01
Treatment (T)	535.56**	≤ 0.01	391.66**	≤ 0.01
Sampling time (S)	169.28**	≤ 0.01	136.27**	≤ 0.01
Cultivar × treatment	1.56	0.22	29.43**	≤ 0.01
C × S	8.62**	≤ 0.01	8.41**	≤ 0.01
T × S	203.98**	≤ 0.01	166.9**	≤ 0.01
C × T × S	3.29*	0.03	6.73**	≤ 0.01

* and ** indicate significant differences at the *P ≤* 0.05 and *P ≤* 0.01 probability levels, respectively.

**Figure 1:**
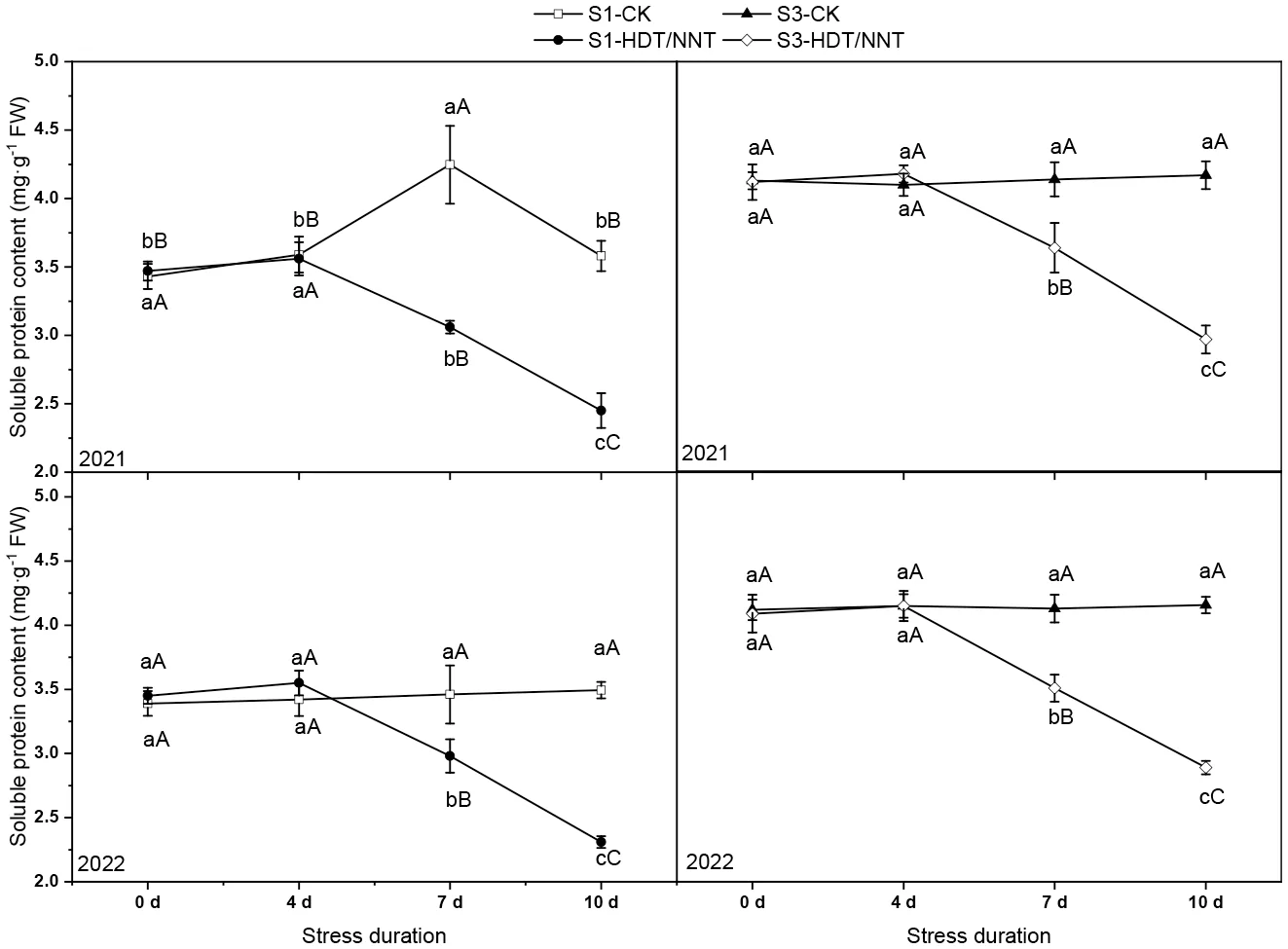
Effects of high-temperature stress under day/night temperature fluctuation on the square soluble protein content in 2021 and 2022. Data are presented as mean ± SD. Error bars represent standard deviation. FW means fresh weight. S1 and S3 represent cultivars ‘Sikang 1’ and ‘Sikang 3’, respectively. CK represents the control treatment; HDT/NNT represents the fluctuating day/night temperature treatment with high daytime temperature/normal night temperature (38/27 °C). The ANOVA results (*F* values and *P* values) are shown in **Table 5**. The significance of the differences between different duration was tested with LSD, and the results were represented by different lowercase and uppercase letters. Pairwise comparisons between different durations were performed using least significant difference (LSD) test at *P* ≤ 0.05 and *P* ≤ 0.01. Different lowercase and uppercase letters represent significantly difference between duration at *P* ≤ 0.05 and *P* ≤ 0.01 respectively under the same treatment for the same cultivar.

In 2021, no significant differences in squares soluble protein contents were detected in S1 under the control treatment, and the same trend was found for S3. While when S1 and S3 were exposed to HDT/NNT, the soluble protein contents stayed stable for no more than 7 days, and once the duration of stress exceeded 7 days, it showed a significant reduction and the longer the duration, the greater the reduction was (**Figure 1**; **Table 5**). In 2021, in comparison with the control treatment, the reduction in soluble protein content was 28.00% and 31.56% on the 7th day and 10th day, respectively, for S1, and they were 12.08% and 28.78% for S3, respectively. In 2022, the reduction on the 7th day was 13.87% and 33.81% for S1, and 15.01% and 30.53% for S3, respectively.

**Table 5:** The ANOVA results (*F* values and *P* values) of square soluble protein content.

Statistics	2021				2022			
	S1-CK	S1-HDT/NNT	S3-CK	S3-HDT/NNT	S1-CK	S1-HDT/NNT	S3-CK	S3-HDT/NNT
*F* value	11.91**	62.2**	0.79	57.48**	0.25	139.71**	0.16	74.14**
*P* value	0.01	≤ 0.01	0.54	≤ 0.01	0.86	≤ 0.01	0.92	≤ 0.01

** indicates significant difference at the P ≤ 0.01 probability level.

**Figure 2:**
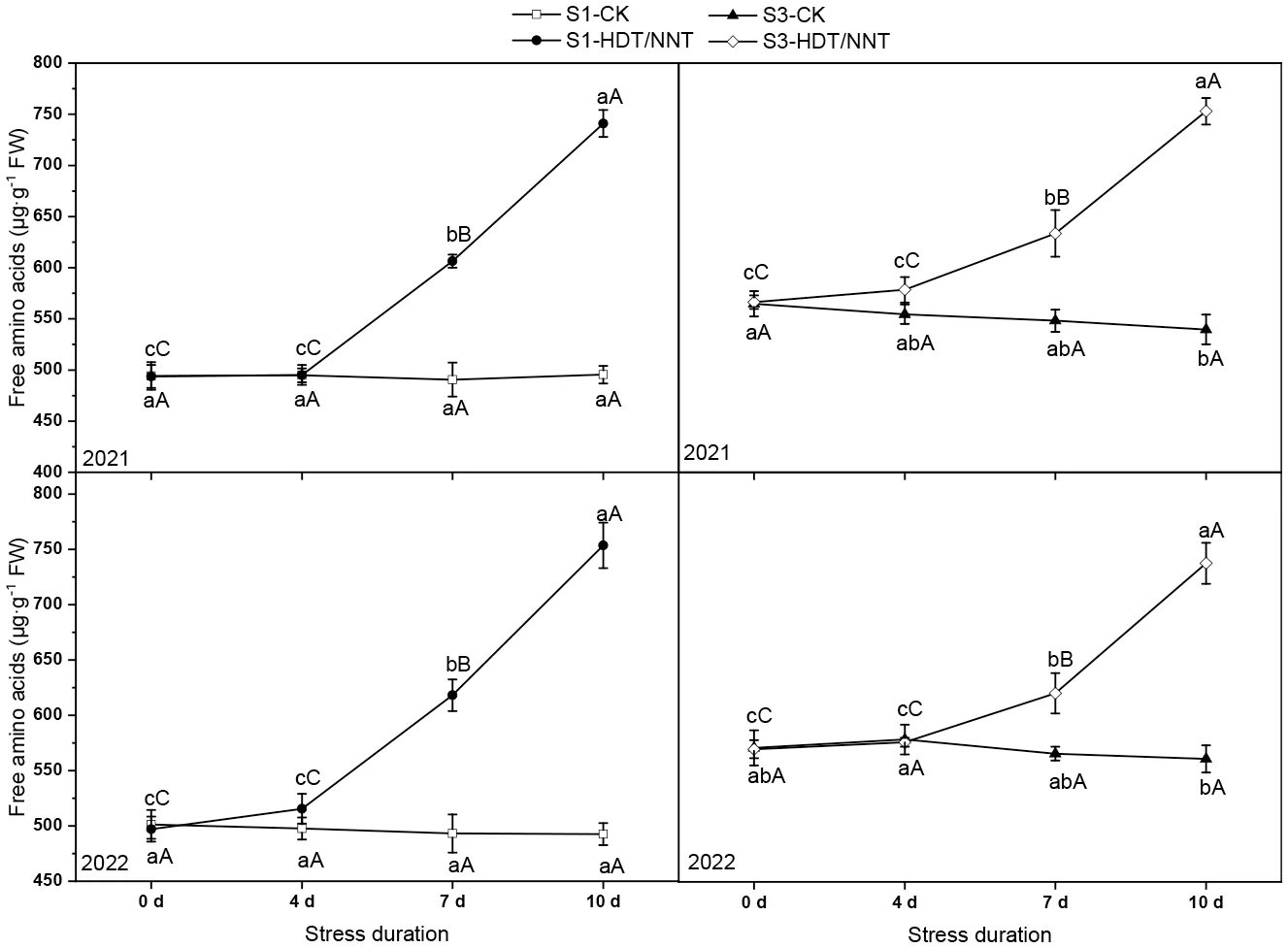
Effects of high-temperature stress under day/night temperature fluctuation on the square free amino acid content in 2021 and 2022. Data are presented as mean ± SD. Error bars represent standard deviation. FW means fresh weight. S1 and S3 represent cultivars ‘Sikang 1’ and ‘Sikang 3’, respectively. CK represents the control treatment; HDT/NNT represents the fluctuating day/night temperature treatment with high daytime temperature/normal night temperature (38/27 °C). The ANOVA results (*F* values and *P* values) are shown in **Table 6**. The significance of the differences between different duration was tested with LSD, and the results were represented by different lowercase and uppercase letters. Pairwise comparisons between different durations were performed using least significant difference (LSD) test at *P* ≤ 0.05 and *P* ≤ 0.01. Different lowercase and uppercase letters represent significantly difference between duration at *P* ≤ 0.05 and *P* ≤ 0.01 respectively under the same treatment for the same cultivar.

There were no significant variations in the square free amino acid contents detected in both S1 and S3 under the control treatment. Under HDT/NNT stress, the free amino acid contents did not increase remarkably until the duration of stress exceeded 7 days (**Figure 2**; **Table 6**). In 2021, on the 7th day and 10th day, the free amino acid contents increased by 23.60% and 49.55% respectively for S1 compared with the control treatment and increased 15.58% and 39.56% respectively for S3 compared with the control treatment. The increase in 2022 was 25.35% and 53.01% for S1, and 9.65% and 31.51% for S3, respectively.

**Table 6:** The ANOVA results (*F* values and *P* values) of square free amino acid content.

Statistics	2021				2022			
	S1-CK	S1-HDT/NNT	S3-CK	S3-HDT/NNT	S1-CK	S1-HDT/NNT	S3-CK	S3-HDT/NNT
*F* value	0.08	346.82**	2.43	91.59**	0.33	143.89**	1.93	133.48**
*P* value	0.97	≤ 0.01	0.16	≤ 0.01	0.8	≤ 0.01	0.23	≤ 0.01

** indicates significant difference at the P ≤ 0.01 probability level.

### 3.3 Response of GOT and GPT activities to high temperature

Three-way ANOVA showed that GOT activity was significantly affected by cultivar, temperature treatment, and sampling time in both 2021 and 2022 (*P ≤* 0.01; **Table 7**). Importantly, a strong Treatment × Sampling time interaction was observed in both years (*P ≤* 0.01), showing that the temperature effect was strongly time-dependent. Neither Cultivar × Treatment nor Cultivar × Sampling time interactions were significant in either year (*P* > 0.05). The three-way interaction was non-significant in both years (*P* > 0.05), indicating that the temporal response of GOT activity to high-temperature stress was highly consistent across cultivars.

**Table 7:** Three-way ANOVA of cultivar, treatment, and sampling time on square GOT activity in 2021 and 2022.

Source of variation	2021		2022	
	*F* value	*P* value	*F* value	*P* value
Cultivar (C)	215.16**	≤ 0.01	150.37**	≤ 0.01
Treatment (T)	133.28**	≤ 0.01	190.61**	≤ 0.01
Sampling time (S)	55.84**	≤ 0.01	102.52**	≤ 0.01
Cultivar × Treatment	0.22	0.65	1.46	0.24
C × S	0.82	0.49	0.49	0.69
T × S	44.74**	≤ 0.01	82.11**	≤ 0.01
C × T × S	1.21	0.32	0.02	1.00

** indicates significant difference at the P ≤ 0.01 probability level.

Consistent with the GOT results, GPT activity was significantly affected by all three main factors in both 2021 and 2022 (*P ≤* 0.01; **Table 8**). Importantly, a strong Treatment × Sampling time interaction was observed in both years (*P ≤* 0.01), showing that the temperature effect was strongly time-dependent. A significant Cultivar × Treatment interaction was detected in both years (*P ≤* 0.05), whereas the Cultivar × Sampling time interaction was non-significant (*P* > 0.05). The three-way interaction was non-significant in both years (*P* > 0.05), indicating that the temporal response of GPT activity to high-temperature stress was consistent across cultivars.

**Table 8:** Three-way ANOVA of cultivar, treatment, and sampling time on square GPT activity in 2021 and 2022.

Source of variation	2021		2022	
	*F* value	*P* value	*F* value	*P* value
Cultivar (C)	429.58**	≤ 0.01	511.29**	≤ 0.01
Treatment (T)	272.74**	≤ 0.01	403.14**	≤ 0.01
Sampling time (S)	86.08**	≤ 0.01	124.54**	≤ 0.01
Cultivar × treatment	4.28*	0.05	16.33**	≤ 0.01
C × S	0.93	0.44	2.44	0.08
T × S	79.36**	≤ 0.01	127.26**	≤ 0.01
C × T × S	1.00	0.41	2.27	0.10

* and ** indicate significant differences at the *P ≤* 0.05 and *P ≤* 0.01 probability levels, respectively.

**Figure 3:**
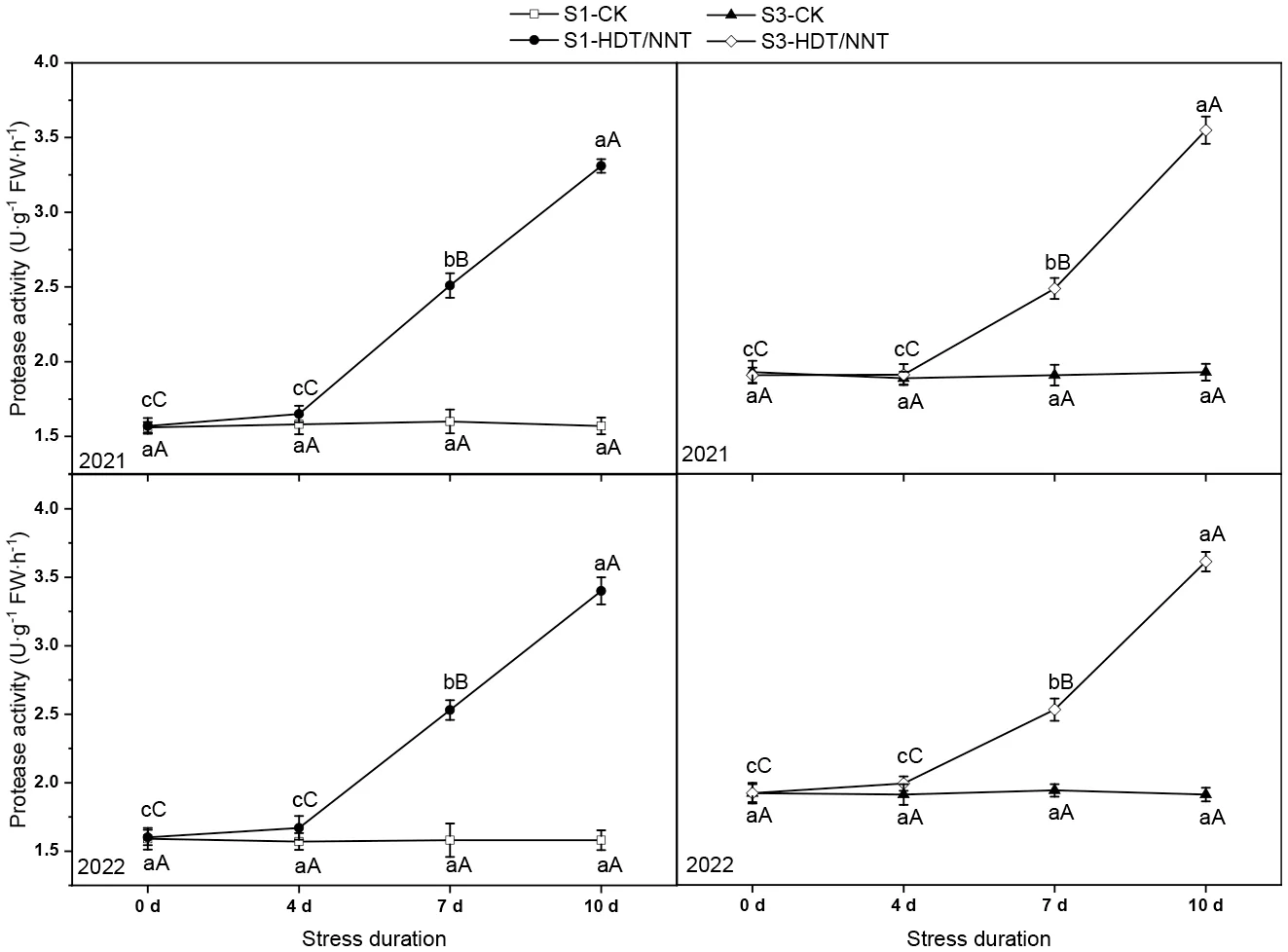
Effects of high-temperature stress under day/night temperature fluctuation on the square GOT activity in 2021 and 2022. Data are presented as mean ± SD. Error bars represent standard deviation. FW means fresh weight. S1 and S3 represent cultivars ‘Sikang 1’ and ‘Sikang 3’, respectively. CK represents the control treatment; HDT/NNT represents the fluctuating day/night temperature treatment with high daytime temperature/normal night temperature (38/27 °C). The ANOVA results (*F* values and *P* values) are shown in **Table 9**. The significance of the differences between different durations was tested with LSD, and the results were represented by different lowercase and uppercase letters. Pairwise comparisons between different durations were performed using least significant difference (LSD) test at *P* ≤ 0.05 and *P* ≤ 0.01. Different lowercase and uppercase letters represent significant difference between different durations at *P* ≤ 0.05 and *P* ≤ 0.01 respectively under the same treatment for the same cultivar.

For both S1 and S3, the square GOT activity did not vary significantly under the control treatment throughout the experiment in both 2021 and 2022. However, when the two cultivars were subjected to HDT/NNT stress for more than 7 days, there was a significant decrease in the square GOT activity (**Figure 3**; **Table 9**). In 2021, on the 7th day, HDT/NNT led to the GOT activity decreasing to 82.33% for S1 and 87.44% for S3 compared with CK, respectively. On the 10th day, GOT activity decreased to 73.40% for S1 and 72.86% for S3 respectively compared with CK in 2021. Similarly, in 2022, HDT/NNT led to a reduction of 17.67% for S1 and a reduction of 12.56% for S3 compared with CK on the 7th day; on the 10th day, the GOT activity decreased to 53.64% for S1 and 61.52% for S3 compared with CK.

**Table 9:** The ANOVA results (*F* values and *P* values) of square GOT activity.

Statistics	2021				2022			
	S1-CK	S1-HDT/NNT	S3-CK	S3-HDT/NNT	S1-CK	S1-HDT/NNT	S3-CK	S3-HDT/NNT
*F* value	0.93	56.74**	0.56	53.14**	0.4	71.61**	2.16	87.57**
*P* value	0.48	≤ 0.01	0.66	≤ 0.01	0.76	≤ 0.01	0.19	≤ 0.01

** indicates significant difference at the P ≤ 0.01 probability level.

**Figure 4:**
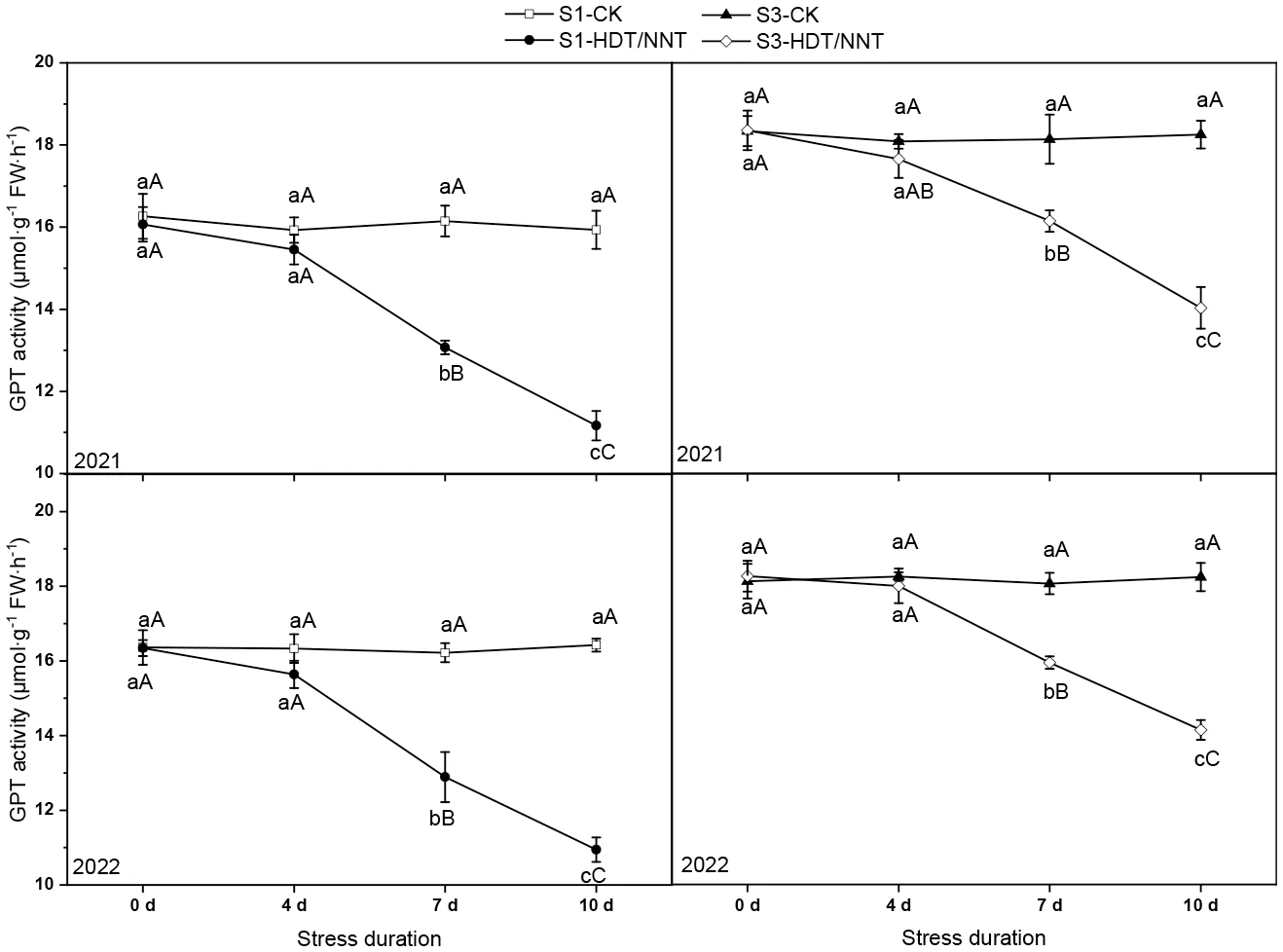
Effects of high-temperature stress under day/night temperature fluctuation on the square GPT activity in 2021 and 2022. Data are presented as mean ± SD. Error bars represent standard deviation. FW means fresh weight. S1 and S3 represent cultivars ‘Sikang 1’ and ‘Sikang 3’, respectively. CK represents the control treatment; HDT/NNT represents the fluctuating day/night temperature treatment with high daytime temperature/normal night temperature (38/27 °C). The ANOVA results (*F* values and *P* values) are shown in **Table 10**. The significance of the differences between different duration was tested with LSD, and the results were represented by different lowercase and uppercase letters. Pairwise comparisons between different durations were performed using least significant difference (LSD) test at *P* ≤ 0.05 and *P* ≤ 0.01. Different lowercase and uppercase letters represent significant difference between duration at *P* ≤ 0.05 and *P* ≤ 0.01 respectively under the same treatment for the same cultivar.

As to the GPT activity, the value did not vary significantly under the control treatment throughout the experiment for both two cultivars in both 2021 and 2022 (**Figure 4**; **Table 10**). HDT/NNT resulted in a reduction of 19.07% for S1 and a reduction of 10.97% for S3 compared with the control treatment, on the 7th day in 2021. Moreover, on the 10th day, the decrement was 29.88% for S1 and 23.12% for S3. In 2022, HDT/NNT led to the GPT activity reducing to 80.27% and 88.26% for S1 and S3 respectively on the 7th day and to 66.63% and 77.51% for S1 and S3 respectively on the 10th day.

**Table 10:** The ANOVA results (*F* values and *P* values) of square GPT activity.

Statistics	2021				2022			
	S1-CK	S1-HDT/NNT	S3-CK	S3-HDT/NNT	S1-CK	S1-HDT/NNT	S3-CK	S3-HDT/NNT
*F* value	0.47	136.33**	0.22	43.6**	0.21	121.62**	0.18	130.58**
*P* value	0.72	≤ 0.01	0.88	≤ 0.01	0.89	≤ 0.01	0.9	≤ 0.01

** indicates significant difference at the P ≤ 0.01 probability level.

### 3.4 Response of protease and peptidase activities to high temperature

Three-way ANOVA showed that protease activity was significantly affected by cultivar, temperature treatment, and sampling time in both 2021 and 2022 (*P ≤* 0.01; **Table 11**). Importantly, a strong Treatment × Sampling time interaction was observed in both years (*P ≤* 0.01), showing that the temperature effect was strongly time-dependent. A significant Cultivar × Treatment interaction was detected in both years (*P ≤* 0.01), whereas the Cultivar × Sampling time interaction was significant in 2021 (*P ≤* 0.01) but not in 2022 (*P* > 0.05). The three-way interaction was significant in both years (*P ≤* 0.05), indicating that the time course of protease activation under high-temperature stress exhibited slight genotypic sensitivity differences.

**Table 11:** Three-way ANOVA of cultivar, treatment, and sampling time on square protease activity in 2021 and 2022.

Source of variation	2021		2022	
	*F* value	*P* value	*F* value	*P* value
Cultivar (C)	212.4**	≤ 0.01	175.93**	≤ 0.01
Treatment (T)	1,094.43**	≤ 0.01	992.81**	≤ 0.01
Sampling time (S)	457.74**	≤ 0.01	378.27**	≤ 0.01
Cultivar × Treatment	12.47**	≤ 0.01	9.37**	≤ 0.01
C × S	5.76**	≤ 0.01	2.80	0.06
T × S	445.73**	≤ 0.01	379.6**	≤ 0.01
C × T × S	3.42*	0.03	3.82*	0.02

* and ** indicate significant differences at the *P ≤* 0.05 and *P ≤* 0.01 probability levels, respectively.

Similarly, peptidase activity was significantly affected by cultivar, temperature treatment, and sampling time in both 2021 and 2022 (*P ≤* 0.01; **Table 12**). Importantly, a strong Treatment × Sampling time interaction was observed in both years (*P ≤* 0.01), showing that the temperature effect was strongly time-dependent. Neither Cultivar × Treatment nor Cultivar × Sampling time interactions were significant in either year (*P* > 0.05). The three-way interaction was non-significant in both years (*P* > 0.05), indicating that the temporal response of peptidase activity to high temperature stress was consistent across cultivars.

**Table 12:** Three-way ANOVA of cultivar, treatment, and sampling time on square peptidase activity in 2021 and 2022.

Source of variation	2021		2022	
	*F* value	*P* value	*F* value	*P* value
Cultivar (C)	97.58**	≤ 0.01	18.09**	≤ 0.01
Treatment (T)	417.51**	≤ 0.01	253.58**	≤ 0.01
Sampling time (S)	221.45**	≤ 0.01	151.16**	≤ 0.01
Cultivar × Treatment	1.76	0.19	0.7	0.41
C × S	1.80	0.17	0.82	0.49
T × S	196.48**	≤ 0.01	129.82**	≤ 0.01
C × T × S	1.87	0.16	0.96	0.42

** indicates significant difference at the P ≤ 0.01 probability level.

**Figure 5:**
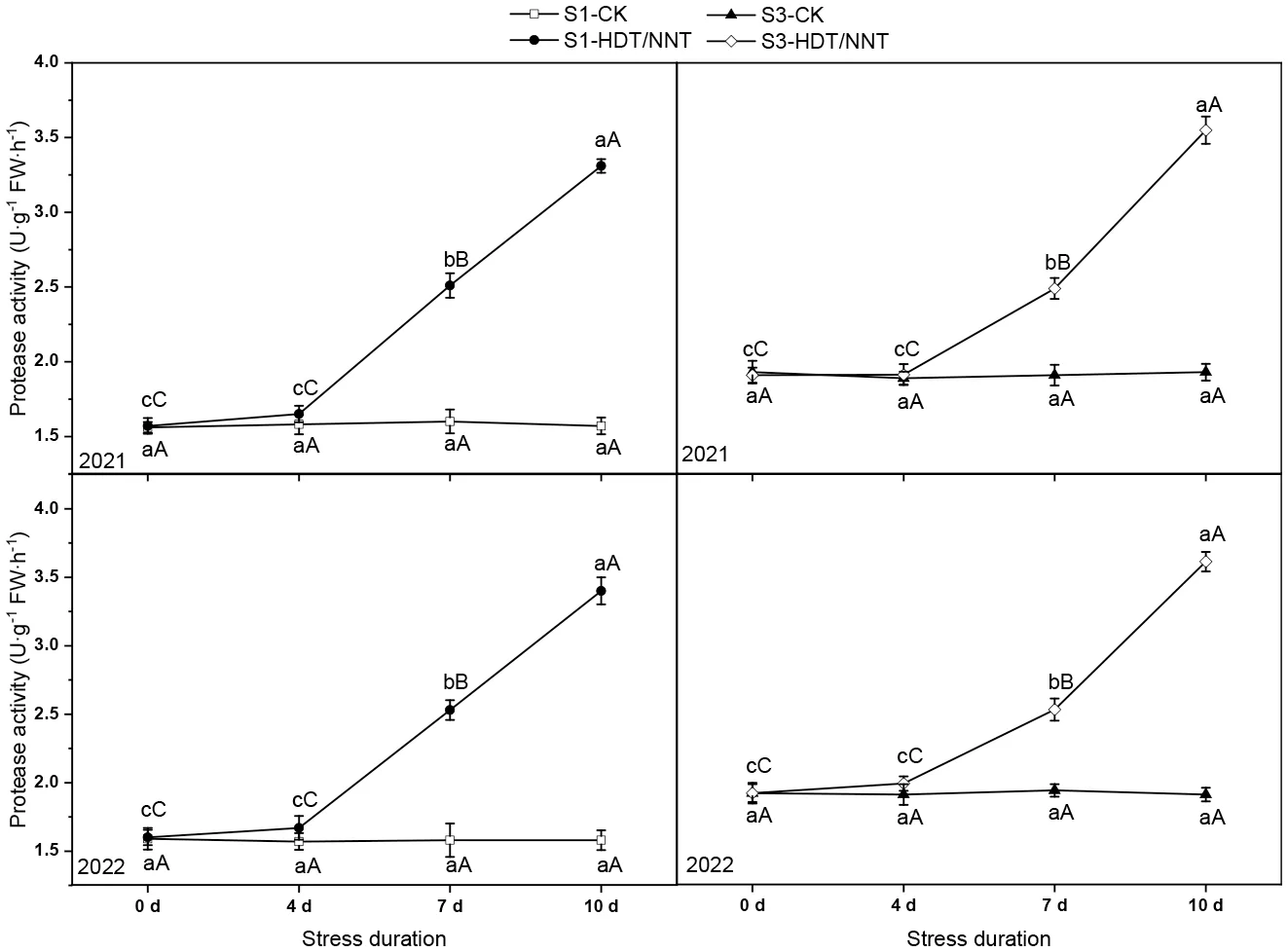
Effects of high-temperature stress under day/night temperature fluctuation on the square protease activity in 2021 and 2022. Data are presented as mean ± SD. Error bars represent standard deviation. FW means fresh weight. S1 and S3 represent cultivars ‘Sikang 1’ and ‘Sikang 3’, respectively. CK represents the control treatment; HDT/NNT represents the fluctuating day/night temperature treatment with high daytime temperature/normal night temperature (38/27 °C). The ANOVA results (*F* values and *P* values) are shown in **Table 13**. The significance of the differences between different duration was tested with LSD, and the results were represented by different lowercase and uppercase letters. Pairwise comparisons between different durations were performed using least significant difference (LSD) test at *P* ≤ 0.05 and *P* ≤ 0.01. Different lowercase and uppercase letters represent significant difference between duration at *P* ≤ 0.05 and *P* ≤ 0.01 respectively under the same treatment for the same cultivar.

In both 2021 and 2022, no significant differences in squares protease activity were detected in S1 under the control treatment, as well as in S3. However, when the two cultivars suffered from HDT/NNT stress for more than 7 days, the protease activity showed remarkable enhancement and the longer the duration, the greater the increase was (**Figure 5**; **Table 13**). Compared with the control treatment, the protease activity exhibited increase of 56.88% for S1 and 30.37% for S3 on the 7th day in 2022. The enhancement was 60.13% and 30.41% in 2022.

**Table 13:** The ANOVA results (*F* values and *P* values) of square protease activity.

Statistics	2021				2022			
	S1-CK	S1-HDT/NNT	S3-CK	S3-HDT/NNT	S1-CK	S1-HDT/NNT	S3-CK	S3-HDT/NNT
*F* value	0.27	419.42**	0.28	299.62**	0.02	360.81**	0.18	310.42**
*P* value	0.84	≤ 0.01	0.84	≤ 0.01	0.99	≤ 0.01	0.91	≤ 0.01

** indicates significant difference at the P ≤ 0.01 probability level.

**Figure 6:**
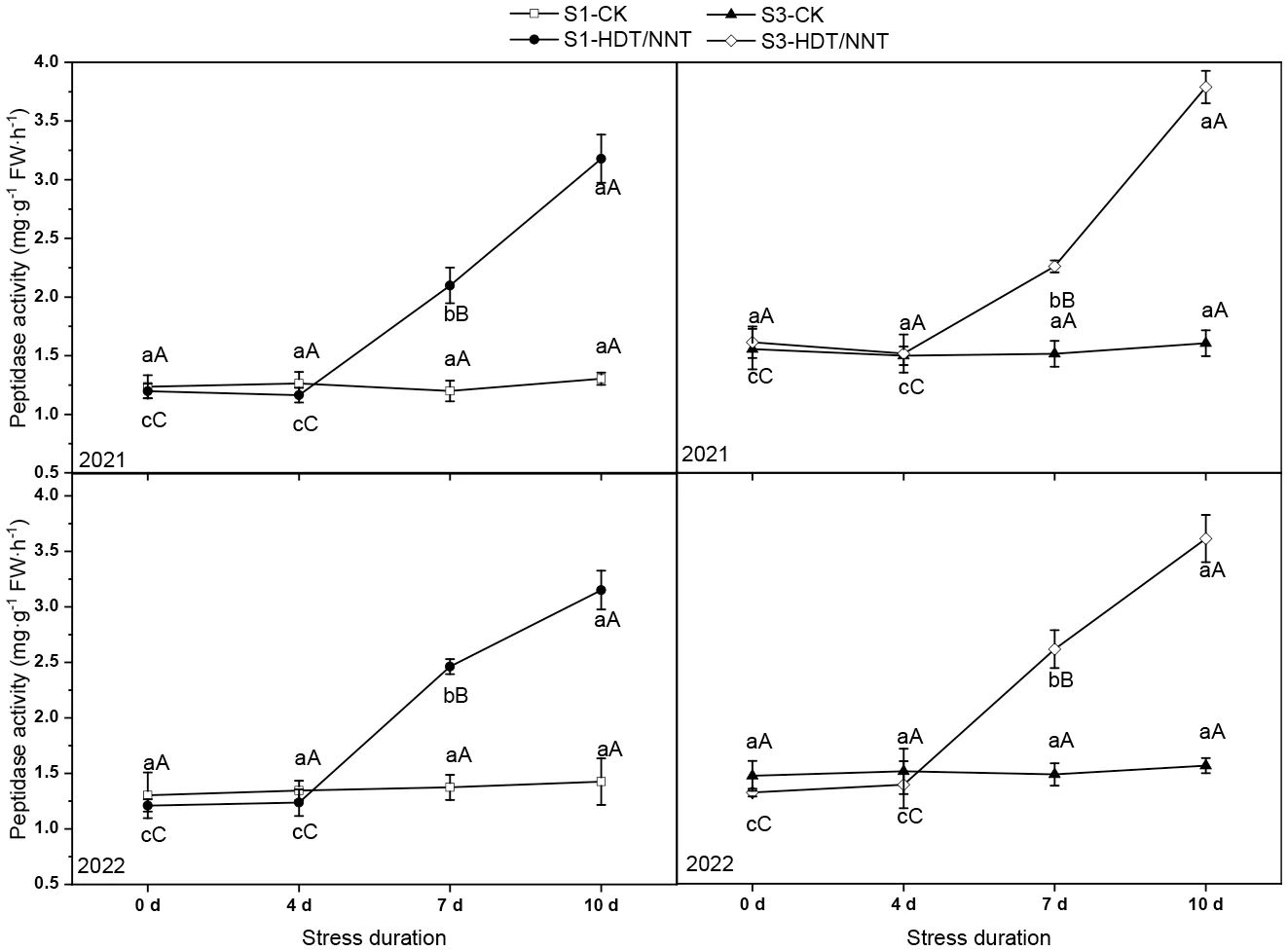
Effects of high-temperature stress under day/night temperature fluctuation on the square peptidase activity in 2021 and 2022. Data are presented as mean ± SD. Error bars represent standard deviation. FW means fresh weight. S1 and S3 represent cultivars ‘Sikang 1’ and ‘Sikang 3’, respectively. CK represents the control treatment; HDT/NNT represents the fluctuating day/night temperature treatment with high daytime temperature/normal night temperature (38/27 °C). The ANOVA results (*F* values and *P* values) are shown in **Table 14**. The significance of the differences between different duration was tested with LSD, and the results were represented by different lowercase and uppercase letters. Pairwise comparisons between different durations were performed using least significant difference (LSD) test at *P* ≤ 0.05 and *P* ≤ 0.01. Different lowercase and uppercase letters represent significant difference between duration at *P* ≤ 0.05 and *P* ≤ 0.01 respectively under the same treatment for the same cultivar.

There was no significant variation in the square peptidase activity recorded in both S1 and S3 under the control treatment throughout the experiment in 2021 and 2022. While when they were exposed to HDT/NNT stress, the peptidase activity remained stable within 7 days, and once the duration of stress exceeded 7 days, it showed a significant increase (**Figure 6**; **Table 14**). On the 7th day and 10th day, the peptidase activity increased to 175% and 244.62% respectively for S1 compared with the control treatment and increased to 148.68% and 235.40% respectively for S3 compared with the control treatment. In 2022, the value peptidase activity increased 79.56% for S1 and 75.17% for S3 on the 7th day and 120.28% for S1 and 129.94% for S3 on the 10th day, respectively.

**Table 14:** The ANOVA results (*F* values and *P* values) of square peptidase activity.

Statistics	2021				2022			
	S1-CK	S1-HDT/NNT	S3-CK	S3-HDT/NNT	S1-CK	S1-HDT/NNT	S3-CK	S3-HDT/NNT
*F* value	0.65	121.44**	0.59	170.29**	0.27	297.03**	0.21	101.62**
*P* value	0.61	≤ 0.01	0.65	≤ 0.01	0.84	≤ 0.01	0.88	≤ 0.01

** indicates significant difference at the P ≤ 0.01 probability level.

### 3.5 Correlation between Cry1Ac endotoxin concentration and indicators of nitrogen metabolism

**Figure 7:**
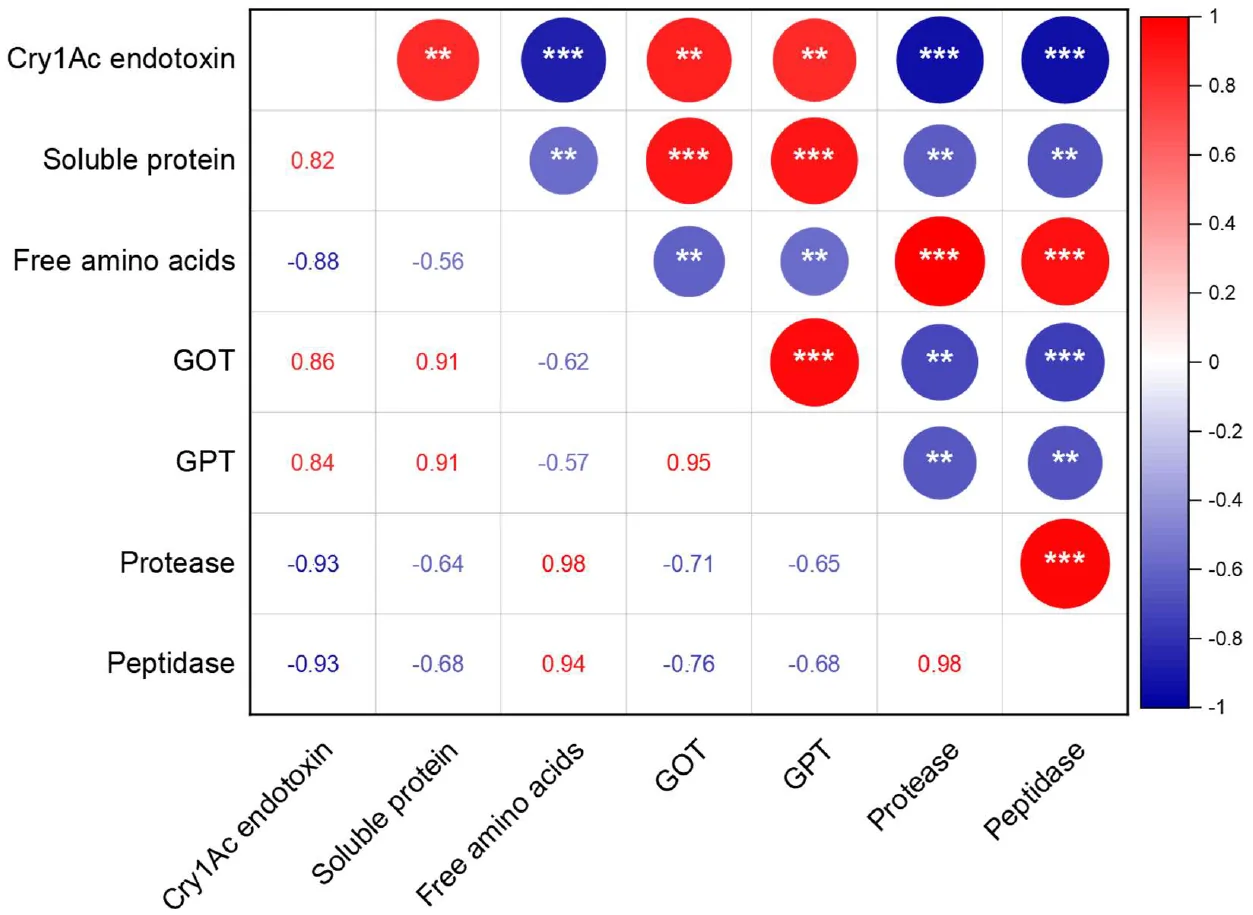
Correlations between Cry1Ac endotoxin content and nitrogen metabolism related parameters. *, **, and *** represent the significance levels of 5%, 1%, and 0.1%, respectively.

The square Cry1Ac endotoxin concentration was significantly positively correlated with soluble protein content, GOT activity, and GPT activity, and the values were found to be 0.82**, 0.86**, and 0.84**, respectively. Furthermore, it was found that there was a significant negative correlation between the square Cry1Ac endotoxin concentration and free amino acid content, protease activity, and peptidase activity, with the values to be –0.88**, –0.93***, and –0.93***, respectively (**Figure 7**).

## 4 Discussion

### 4.1 High-temperature stress under day/night temperature fluctuation lasting more than 7-day reduced Cry1Ac endotoxin concentration in squares

Multiple studies have shown that high temperatures cause a sharp decrease in Cry1Ac endotoxin content in Bt cotton and that the response to high temperatures varies between growth stages and organs (Xia and Guo, 2004; Zhang et al., 2021; Jehangir, 2023). Chen et al. (2005) reported that when plants at different growth stages were exposed to high temperatures of 38 °C for 24–48 h, there were no significant changes in Cry1Ac endotoxin concentration of main stem leaves at peak square and peak flower stage, but it appeared to be significantly decreased at boll setting stage. Dong and Li (2007) clarified that 37 °C was the critical high temperature that caused the significant decrease in Cry1Ac endotoxin concentration in leaves, whereas Chen et al. (2014) demonstrated that the critical high temperature that caused the significant decrease in Cry1Ac endotoxin concentration in squares was 38 °C. The difference may be attributed to the different tolerances of various organs to high temperatures. Additionally, high temperature and the interaction of different stresses affected the Cry1Ac endotoxin concentration of Bt cotton to different extents. For example, Chen et al (2012a) found that extreme low or high air relative humidity (50% and 90%) led to a relatively faster and larger decline in Cry1Ac endotoxin concentration in leaves under high-temperature stress, compared with normal relative humidity (70%). Jin et al. (2024) exposed that drought stress further exacerbates the reducing effect of high temperature on Cry1Ac endotoxin concentration in Bt cotton. In addition, the performance of conventional and hybrid cultivars under high-temperature stress was also different. Liu et al. (2022) observed that after termination of high-temperature stress at 38 °C for 72 h and 96 h, it took 48 and 72 h, respectively, for Cry1Ac endotoxin concentration in squares of conventional and hybrid cultivars to recover to the normal level.

In this study, we observed that the Cry1Ac endotoxin concentration of squares at the peak square stage showed a significant reduction in response to high-temperature stress under day/night temperature fluctuations, which was consistent with the results of previous studies (Ahmad et al., 2021). However, in our study, the Cry1Ac endotoxin concentration of squares did not vary significantly when exposed to stress for up to 7 days. Instead, a significant decrease was detected when the stress was sustained for 7 days and above, which was not in agreement with the significant decrease being recorded after 24 h of high-temperature stress as reported by Chen et al. (2014). We hypothesized that this significant delay in Cry1Ac endotoxin concentration reduction was mainly owing to the day/night temperature fluctuation of high-temperature stress in this experiment, i.e., Bt cotton plants were exposed to high temperature of 38 °C during the daytime, whereas at night they were at a moderate temperature of 27 °C. Under daytime high-temperature stress, plants were likely to show the same decreased trends in Cry1Ac endotoxin concentration as under constant high-temperature stress, but moderate nighttime temperatures attenuated diurnal stress intensity, allowing temporary physiological compensation, which enable a temporary physiological equilibrium between baseline protein synthesis and degradation. Furthermore, our previous research showed that the time required for Cry1Ac endotoxin concentration to recover to normal levels was inversely proportional to the duration of the high-temperature stress exposure (Chen et al., 2013). Instead of claiming an immediate nighttime recovery, our 11:00 data point reflects a day–night physiological compensation under the 38/27 °C regime. Unlike constant high-temperature stress (e.g., 38/38 °C), which rapidly impairs nitrogen metabolism and Cry1Ac levels within 24 h (Chen et al., 2012b), the moderate 27 °C night in our setup helps buffer daytime thermal damage. Furthermore, because Cry1Ac expression is driven by the constitutive CaMV 35S promoter and the protein turns over relatively slowly (Olsen et al., 2005), the stable toxin and enzyme concentrations measured at 11:00 represent the net balance between daytime stress and nighttime recovery, rather than short-term circadian shifts. Nevertheless, with prolonged stress exposure, accumulated damage eventually disrupts this delicate balance, resulting in a marked decline in Cry1Ac endotoxin concentration. As demonstrated in this study, a significant decrease was observed after 7 days of continuous stress. It is worth noting that the 7-day threshold observed in this study should be interpreted as a biological tipping point representing the limit of nocturnal compensatory capacity, rather than a coincidental or abrupt temporal trigger. During the first 4 days of fluctuating stress, cotton squares successfully compensated for daytime thermal damage through nighttime compensation, keeping the net Cry1Ac concentration unchanged. However, between day 4 and day 7, cumulative thermal stress exceeded this physiological tolerance threshold. Objectively speaking, because our sampling schedule evaluated physiological traits at 0, 4, 7, and 10 days, our data reliably define the day 4 to day 7 window as the critical transition period for metabolic equilibrium. Based on our experimental observations, day 7 represents a definitive biological tipping point where nighttime compensation capacity is completely exhausted, causing a system-wide collapse in protein synthesis and endotoxin stability. Whether this qualitative physiological inversion initiates earlier—specifically on day 5 or day 6—remains to be elucidated and warrants further daily-resolution investigations. Nevertheless, from an ecological and agronomic perspective, day 7 serves as a robust and practical benchmark for sustained heatwave monitoring in field environments. Given the natural day/night temperature variations, cotton plants under field cultivation typically experience a cycle of daytime high-temperature stress followed by nighttime moderate temperature, which is in accordance with the experimental design applied in this study. Consequently, our findings provided a more realistic representation of how the Cry1Ac endotoxin concentration responds to high-temperature stress in real field environments. More importantly, in China’s cotton-growing regions, high temperatures typically persist from late June (the square stage) through mid-August (the boll-setting stage), generally ranging between 36 °C and 40 °C for 6 to 10 consecutive days (Chen et al., 2005). Hence, during actual production, particular attention must be paid to prolonged high temperatures exceeding 7 days during the square stage, and timely management measures should be implemented.

Some strategies can be considered to mitigate long-term high-temperature stress. For example, exogenous regulators such as salicylic acid and mepiquat chloride is beneficial to alleviating high-temperature stress in cotton plants, so if a high-temperature event exceeding 7 days is predicted, foliar application of 200 mg·L^-^¹ salicylic acid or 20 mg·L^-^¹ mepiquat chloride is recommended for the plants (Zhao et al., 2025). Additionally, high-temperature stress in a field environment is often accompanied by drought stress. Therefore, when a high-temperature event exceeding 7 days occurs, timely irrigation is necessary to prevent the interaction between high temperature and drought stress, which could lead to a more severe reduction in Cry1Ac endotoxin concentration in squares.

### 4.2 The weakening of protein synthesis capacity and the intensification of protein degradation processes were the main reasons for the decline in Cry1Ac endotoxin concentration

The Cry1Ac endotoxin content is a soluble protein (Chen et al., 2005), and many studies have clarified that its content in Bt cotton is closely related to protein metabolism under various adversity stresses (Luo et al., 2017; Chen et al., 2019; Liu et al., 2022). In this study, it was noticed that when there was a significant decrease in Cry1Ac endotoxin content in squares, the soluble protein content, GOT activity, and GPT activity decreased as well; in contrast, free amino acid content, protease activity, and peptidase activity in squares showed increasing trends. Similarly, correlation analyses showed that there existed significant positive correlations between square Cry1Ac endotoxin concentration and soluble protein content, GOT activity, and GPT activity, whereas it was significantly negatively correlated with free amino acid content, protease activity, and peptidase activity. Furthermore, this correlation analysis elucidates the complex regulatory network of Cry1Ac endotoxin expression under fluctuating high-temperature stress, characterized by distinct synergistic and antagonistic interactions among nitrogen metabolism indicators. Specifically, GOT and GPT activities exhibit a strong synergistic relationship with soluble protein content, collectively driving the nitrogen assimilation and protein synthesis pathways that maintain Cry1Ac endotoxin concentration levels. Conversely, these synthesis-related indicators are strongly antagonized by the protein degradation pathway, which is governed by protease and peptidase. Under short-term stress (≤7 days), the synergistic synthesis pathway effectively counterbalances the antagonistic degradation pathway, buffered by the day/night physiological compensatory equilibrium. However, as stress accumulation progresses from day 4 to day 7, the squares undergo a qualitative physiological transition. This is not an isolated change in Cry1Ac alone, but a coordinated system-wide metabolic inversion: the synthesis pathway (GOT/GPT activities and soluble protein) is substantially suppressed, whereas the antagonistic degradation pathway (protease and peptidase activities) becomes overwhelmingly dominant. By the 7-day mark, this dynamic equilibrium completely collapses, hydrolyzing proteins into free amino acids and leading to the observed drop in Cry1Ac concentration. The antagonistic degradation forces become overwhelmingly dominant, hydrolyzing proteins into free amino acids. This physiological shift explains the simultaneous increase in free amino acids content and the rapid decrease of Cry1Ac endotoxin concentration observed in this study. The above results indicated that when the duration of high-temperature stress with day/night temperature fluctuation did not exceed 7 days, the nighttime environment with normal temperatures allowed protein synthesis and degradation in the squares to reach a dynamic equilibrium, which was manifested by the Cry1Ac endotoxin concentration remaining stable. Once the duration of stress exceeded 7 days, however, this equilibrium could no longer be maintained, and the protein synthesis capacity of squares was rapidly weakened and protein degradation was sharply increased, which ultimately combined to lead to a significant reduction in Cry1Ac endotoxin concentration.

### 4.3 Methodological considerations, agronomic extrapolation, and future perspectives

Prior to the high-temperature treatments, all plants were grown in a transparent glass greenhouse under natural solar irradiance to ensure normal vegetative and reproductive development. During the subsequent stress exposure, the irradiance used in our artificial climate chamber (200 μmol m^-^² s^-^¹) was relatively low compared with typical summer solar radiation in the field, where cotton often experiences levels above 1,000 μmol m^-^² s^-^¹. Because nitrogen assimilation and protein synthesis depend on carbon skeletons, ATP, and NADPH from photosynthetic carbon assimilation, this low light intensity likely led to lower overall carbon-nitrogen metabolism rates and a lower baseline of Cry1Ac endotoxin accumulation than in field-grown plants. However, since both the control and high-temperature treatments received the same irradiance, the metabolic changes and Cry1Ac differences observed in this study were driven primarily by the day/night temperature fluctuations.

Under high solar irradiance in the field, cotton plants accumulate more carbohydrates and energy, which provides more substrates for nitrogen assimilation (supported by GOT and GPT activities) and nighttime physiological buffering. However, because light and temperature closely interact to regulate plant metabolism, it remains uncertain whether the day/night compensatory equilibrium observed under our 38/27 °C regime applies identically to field conditions. We hypothesize that this compensatory mechanism still operates in the field—and may even be stronger—but this requires further verification under natural light conditions.

In addition to environmental irradiance, methodological considerations regarding sampling resolution also warrant evaluation. Sampling at a single daytime point (11:00 a.m.) provided a standardized benchmark to evaluate peak carbon-nitrogen metabolism and protein stability while controlling for circadian noise. However, this static approach reflects the net outcome over the day/night cycle rather than real-time nighttime dynamics. Consequently, the proposed day/night compensatory balance remains a plausible hypothesis supported by our metabolic data. To thoroughly dissect this compensatory equilibrium across both agronomic and molecular dimensions, future studies should combine experiments under realistic field light conditions with frequent nighttime sampling, transcriptomic profiling across time points, and isotopic tracing.

## 5 Conclusion

In this study, we investigated the response of Bt squares Cry1Ac endotoxin concentration to a type of high-temperature stress that more closely resembles the high-temperature stress in a field environment, that is, under day/night temperature fluctuation. The results showed that high-temperature stress with a short period of day/night temperature fluctuation did not significantly affect the Cry1Ac endotoxin concentration of squares, whereas once the duration of the stress exceeded 7 days, the Cry1Ac endotoxin concentration underwent a remarkable decrease, and the longer the duration of the stress, the greater the decrease was. For example, the reduction was 14.18% and 30.88% on the 7th day and 10th day for S1, and 11.32% and 25.21% on the 7th day and 10th day for S3, in 2021. Hence, based on the coordinated changes in nitrogen metabolism, we propose that normal nighttime temperatures provide temporary physiological compensation that delays high-temperature-induced metabolic imbalance against daytime high-temperature stress. This nighttime relief potentially facilitates a temporary dynamic balance between Cry1Ac synthesis and degradation. However, further studies are needed, as direct molecular evidence, such as protein turnover rates and specific gene expression profiling, is required to fully validate this mechanistic hypothesis. Nevertheless, this study demonstrate that the accumulated damage caused by HDT/NNT treatment exceeding 7 days at the peak square stage ultimately disrupts the metabolic compensation, resulting in a significant decrease in Cry1Ac endotoxin concentration.

## Data Availability

The original contributions presented in the study are included in the article/supplementary material. Further inquiries can be directed to the corresponding author.
